# Validation of the Turkish version of the Developmental Behavior Checklist: a comprehensive tool for assessing emotional, behavioral, and autism spectrum disorders in children and adolescents with intellectual disabilities

**DOI:** 10.3389/fpsyt.2025.1579629

**Published:** 2025-04-28

**Authors:** Sabide Duygu Uygun, Merve Çıkılı Uytun, Hande Konşuk Ünlü, Ayşegül Akgül Doğru, Serpil Aktaş Altunay, Didem Behice Öztop, Birim Günay Kılıç, Kerim Munir

**Affiliations:** ^1^ Department of Child and Adolescent Psychiatry, Ankara University School of Medicine, Ankara, Türkiye; ^2^ Institute of Public Health, Hacettepe University, Ankara, Türkiye; ^3^ Department of Child and Adolescent Psychiatry, Bitlis Tatvan State Hospital, Bitlis, Türkiye; ^4^ Department of Statistics, Hacettepe University, Ankara, Türkiye; ^5^ Division of Child and Adolescent Psychiatry, Private Clinic, Ankara, Türkiye; ^6^ Division of Developmental Medicine, Boston Children’s Hospital, Harvard Medical School, Boston, MA, United States

**Keywords:** intellectual disability, intellectual developmental disorder, autism spectrum disorder, children, adolescents

## Abstract

**Introduction:**

The Developmental Behavior Checklist (DBC) is a standardized tool for evaluating emotional and behavioral concerns in children and adolescents with intellectual disabilities (ID) in clinical and research settings. This study aims to validate the Turkish versions of the DBC autism screening algorithm (ASA), parent (P) and teacher (T) forms.

**Methods:**

Parents and teachers of 312 children and adolescents aged 4–17 with ID completed the Turkish versions of the DBC forms and the Strengths and Difficulties Questionnaire (SDQ). The presence of ASD or any other psychiatric disorder was assessed through clinical evaluation based on the Diagnostic and Statistical Manual of Mental Disorders, Fifth Edition, Text Revision (DSM-5-TR) guidelines. Analyses of validity and reliability were conducted to evaluate the internal consistency, sensitivity, specificity, area under the curve (AUC), convergent validity, inter-rater reliability, and test-retest reliability.

**Results:**

Among the participants, 30.6% (n=70) had a psychiatric comorbid diagnosis according to the DSM-5-TR. DBC-P exhibited a sensitivity of 64.4%, a specificity of 87.6%, and an AUC of 76%. DBC-T demonstrated a sensitivity of 89.7%, a specificity of 75.8%, and an AUC of 82.7%. Additionally, 23.1% (n=45) were diagnosed with autism spectrum disorder (ASD). The DBC-ASA showed a sensitivity of 58.1%, a specificity of 64.6%, and an AUC of 61.3%. The DBC forms displayed strong internal consistency, robust test-retest reliability, and significant correlation with the SDQ measures. Inter-rater agreement between the DBC-P and DBC-T was low to moderate. A significant difference between parent and teacher assessments highlights the need for multi-informant approaches (p <.001).

**Discussion:**

The DBC-P and DBC-T exhibit high validity and reliability, while the DBC-ASA shows moderate accuracy. This study acts as a valuable resource for clinicians, providing enhanced support for Turkish children and adolescents with ID.

## 1 Introduction

Intellectual disability (ID), also referred to as an intellectual developmental disorder in the Diagnostic and Statistical Manual of Mental Disorders, Fifth Edition, Text Revision (DSM-5-TR) (1), is a neurodevelopmental condition marked by significant limitations in both intellectual functioning and adaptive behavior, that hinder age-appropriate daily life activities (2, 3). Children and adolescents with ID face an increased risk of encountering emotional and behavioral issues, which can significantly affect their quality of life, social functioning, and developmental outcomes. Proper identification and assessment of these issues are essential for guiding interventions; however, the availability of culturally and linguistically appropriate tools is still limited, especially in non-Western contexts. The Developmental Behavior Checklist (DBC) is a widely utilized tool for evaluating emotional and behavioral concerns in individuals with ID. Nevertheless, its applicability and psychometric properties need validation within specific cultural settings to ensure reliability and validity.

Currently, more than half of the population-based cohort of children and adolescents diagnosed with mild ID between the ages of 3 and 15 have been noted to have one or more additional co-occurring mental disorders recorded at adult follow-up, including attention-deficit/hyperactivity disorder (ADHD), autism spectrum disorder (ASD), anxiety, and substance use-related problems (4). Such co-occurring psychiatric disorders further diminish the quality of life for both individuals and families, leading to significant financial burdens on society (5). The clinical characteristics of ID may overlap with or obscure the specific symptoms of psychiatric disorders, making assessment challenging. Children and adolescents with ID also struggle to verbally express their emotions and thoughts compared to their typically developing peers (6). Further, they tend to exhibit poorer executive functions, emotion regulation, and motor coordination, which can lead to hyperactivity, impulsivity, and aggressive behaviors. Others may be shy and withdrawn (7). Additionally, the manifestation of these co-occurring conditions can differ significantly among individuals with ID. Those with severe ID may display stereotyped movements and challenging behaviors directed toward themselves or others (8). Nevertheless, before attributing these symptoms to ID and assisting in treatment planning, it is crucial to carefully monitor co-occurring emotional and behavioral changes over time.

The use of general tools like the Strengths and Difficulties Questionnaire (SDQ) and the Achenbach System of Empirically Based Assessment (ASEBA) Child Behavior Checklist (CBCL) in assessing children and adolescents with ID presents several essential limitations (9, 10). Both SDQ and CBCL are designed for typically developing children and may not be sensitive to the unique cognitive and communicative challenges present in children with ID. As a result, these tools may under-report or fail to capture the full extent of emotional and behavioral difficulties experienced by children with ID. For example, items assessing internalizing or externalizing behaviors may not account for developmental expectations for children with ID, leading to an overestimation or underestimation of symptoms.

Tools like SDQ and CBCL assess broad behavioral domains without considering the specific ways in which children with ID might manifest certain behaviors. Behaviors stemming from cognitive limitations may be misinterpreted as psychiatric symptoms, leading to inaccurate assessments. For example, repetitive, stereotyped behaviors or delayed social skills could be mistakenly attributed to psychiatric disorders when they may be more related to the child’s cognitive functioning.

Among specialized assessment tools developed for intellectual disabilities (ID), only the Turkish version of the Aberrant Behavior Checklist (ABC) has shown validity and reliability (11). The ABC primarily focuses on externalizing or “aberrant” behaviors, such as aggression, irritability, and stereotyped actions, which may overlook significant internal emotional issues that are often present in children with intellectual disabilities. While it helps identify severe behavioral concerns, the ABC is less responsive to the developmental stages of children and adolescents and may fail to adequately consider age-appropriate behaviors in ID. Moreover, the ABC was initially designed for populations with and ASD, concentrating on severe behavioral problems. Consequently, the ABC may be less effective in assessing emotional issues in children with mild to moderate ID.

There is a need for a more comprehensive tool that evaluates a broader range of emotional and behavioral challenges than the ABC, which is appropriate for the developmental levels of children and adolescents with ID. Such tools should assess not only externalizing behaviors (e.g., aggression and hyperactivity) but also internalizing behaviors (e.g., anxiety, depression, withdrawal). To fill this gap, the DBC was first devised to be sensitive to developmental concerns in children and adolescents with ID (12–14). The revised checklist 2 (DBC2) instrument has been used internationally for over 20 years and has demonstrated strong psychometric properties, including validity, reliability, specificity, and sensitivity in samples from Australia, the US, the Netherlands, and Germany (6, 14–19).

## 2 Aim

We aimed to assess the reliability and validity of a Turkish version of the DBC Parent (DBC-P) and Teacher (DBC-T) forms, as well as the DBC Autism Screening Algorithm (DBC-ASA), to provide an accurate evaluation of emotional and behavioral challenges faced by children and adolescents with ID in various settings. By establishing the tool’s reliability and validity within the Turkish context, this research seeks to assist clinicians and researchers in effectively identifying and addressing emotional and behavioral difficulties in the country. Enhanced assessments are expected to lead to more targeted treatment plans and support strategies for children and adolescents with ID who experience emotional and behavioral challenges. The availability of such a measure, with a broader scope and developmental sensitivity, is likely to facilitate future research on the prevalence of emotional and behavioral issues in Turkish children with ID, while guiding resource allocation and intervention strategies tailored to the country’s needs. Having a Turkish version of the DBC would enable better comparative research between Turkey and other nations concerning the prevalence and characteristics of emotional and behavioral problems in children with ID. This can aid policymakers and health professionals in understanding Turkey’s unique requirements and improving services for children and adolescents with ID worldwide.

## 3 Materials and methods

### 3.1 Procedure

Western Psychological Services (WPS) granted research permission to validate the revised DBC in Turkey. Two child and adolescent psychiatrists (SDU and MÇU) translated the parent and teacher forms into Turkish, followed by blind back-translation into English. Discrepancies were corrected by consensus.

Ethical approval was obtained from the ethics review committee at Ankara University Medical School. Children and adolescents with ID (IQ <80) aged 4–17 years, attending the Department of Child and Adolescent Psychiatry, were invited to participate in the study with their parents. Informed consent was obtained from parents.

We collected sociodemographic and clinical data during clinical interviews. Parents filled out the DBC-P and SDQ-Parent Form (SDQ-P). They also received the DBC-T and SDQ-Teacher Form (SDQ-T), to give to the teachers. Teacher forms were collected at a follow-up visit. After completing the scales, children and adolescents were assessed through a clinical evaluation based on the DSM-5-TR to determine whether they had ASD or any psychiatric disorder diagnosis.

### 3.2 Measures

#### 3.2.1 Sociodemographic and clinical data form

The Sociodemographic and Clinical Data Form gathered information about the child’s age, sex, educational status, intellectual disability level, presence of ASD or any other psychiatric disorder diagnosed by clinical evaluation based on the DSM-5-TR, and parents’ ages, educational levels, and family income.

#### 3.2.2 Strengths and difficulties questionnaire

The SDQ, which includes both parent and teacher forms, is used to screen for mental health problems in children and adolescents (20). The forms consist of 25 items that assess both positive and negative behavioral attributes, divided into five subscales: (1) conduct problems, (2) hyperactivity and inattention, (3) emotional symptoms, (4) peer relationship problems, and (5) prosocial behaviors. Each subscale is evaluated individually, and the sum of the first four subscales provides a total difficulties score. Based on the cutoff points, the total scores are classified as Normal/Borderline (16 or below for the parent form and 15 or below for the teacher form) or Abnormal (17–40 for the parent form and 16–40 for the teacher form). A high score in any subscale, except for prosocial behaviors, indicates a potential issue. The Turkish version of the SDQ was valid and reliable, with Cronbach’s alpha coefficient of 0.73 (21).

#### 3.2.3 Developmental behavior checklist

The comprehensive assessment includes parent and teacher forms, which are 96-item and 94-item scales, respectively, used to assess behavioral and emotional problems in children and adolescents aged 4–17 with developmental delays or intellectual disabilities (12, 13). A parent or caregiver completes the DBC-P, while the DBC-T is completed by a teacher or assistant teacher. Both forms encompass five subscales: Disruptive, Self-Absorbed, Communication Disturbance, Anxiety, and Social Relating. The DBC is structured similarly to the CBCL (ASEBA) (22). Each item is rated on a scale of 0, 1, or 2, where “0 = not true as far as you know,” “1 = somewhat or sometimes true,” and “2 = very true or often true.” Total scores of the DBC forms are converted into T-scores. Total T-scores include clinical cutoff points to distinguish individuals with clinically significant emotional and behavioral problems from those without. The cutoff points for the DBC forms closely align with clinical diagnoses (13). In the United States (US) sample, the clinical cutoff point for the total T score of the DBC-P is 53, while it is 48 for the DBC-T (14). Based on the total T-scores, concern ranges are created to reflect levels of concern (14). T-scores below 40 represent “little concern,” scores between 40 and 50 represent “moderate concern,” and scores above 50 represent “serious concern.” The forms are reliable tools with an internal consistency of 0.95 for children and adolescents (14). Twenty-nine items were selected from the DBC-P, and the DBC-ASA was designed to effectively differentiate individuals aged 4–18 with ASD and ID from those with only ID (23). The DBC-ASA has demonstrated good psychometric properties, with an optimal cutoff score of 17 and an area under the ROC curve of 0.80, indicating strong sensitivity and specificity for autism screening (23, 24). These forms can be utilized in clinical practice for both assessment and monitoring of interventions and research studies.

### 3.3 Statistical analyses

Statistical analyses were conducted using IBM SPSS Statistics for Windows, version 26.0, and the validity-reliability metrics were calculated with the ‘caret’ package in R. Descriptive statistics are presented as mean ± standard deviation for continuous variables and as frequency (percentage) for categorical variables. The validity and reliability of the DBC forms were evaluated, and the corresponding criteria were computed. Results are provided with point estimates and 95% confidence intervals (CIs). To ensure that emotional and behavioral symptoms that significantly affect the functioning of children and adolescents with ID are not overlooked, the SDQ was used as a reference scale rather than relying solely on the presence of any psychiatric disorder diagnosis determined through clinical evaluation based on DSM-5-TR. As a result, the validity and reliability analyses of the DBC forms were performed accordingly. The total scores of the SDQ-P and SDQ-T were categorized as either ‘normal/borderline’ (labeled as ‘no comorbidity’) or ‘abnormal’ (labeled as ‘comorbidity present’). The total scores of the DBC forms were converted to T-scores and categorized to indicate the presence or absence of comorbidity according to clinical cutoff points. Metrics such as sensitivity, specificity, positive predictive value (precision), negative predictive value, detection rate, and area under the curve (AUC) were used to assess the validity of the DBC forms. To assess the reliability of the DBC forms, the correct classification rate and the Kappa coefficient were applied. For the DBC-ASA, the presence of an ASD diagnosis based on clinical evaluation was used as the reference, and the analyses were repeated. Correlation parameters for the test-retest reliability of the total and subscales of the DBC-P and DBC-T were also calculated. The convergent validity of the total scores and subscales of the DBC-P and DBC-T was assessed by determining their relationships with the total and subscales of the SDQ-P and SDQ-T using correlation coefficients. The sociodemographic characteristics and clinical variables based on the DBC and SDQ forms in children and adolescents with ID diagnosed with and without any psychiatric comorbidity through clinical evaluation based on DSM-5-TR were compared using the Mann-Whitney U test. A p-value of less than .05 was considered statistically significant.

## 4 Results

The study included 312 participants, of whom 195 (62.5%) were male, with a mean age of 11.91 (SD=4.35) (See **Table 1**). Among the participants, 23.1% (n=45) had a comorbid ASD diagnosis, and 30.6% (n=70) had any other psychiatric comorbidity diagnosis based on the DSM-5-TR. Clinical assessments based on the DBC and SDQ forms revealed over one-third of participants, were categorized as having severe emotional and behavioral problems, particularly in social relationships (see **Table 2**). High rates of comorbid issues were observed, with 29.1% to 46% being affected and 37.9% screened positive for ASD.

**Table 1:** Sociodemographic characteristics of participants.

	Mean / n	SD / %
Age/years	11.91	±4.35
Sex		
Female	117	37.5
Male	195	62.5
Special education/years	6.34	±4.14
ID level		
Mild	231	74
Moderate	51	16.3
Severe	30	9.6
Maternal age/years	40.92	±7.72
Maternal education/years	8.36	±4.31
Paternal age/years	44.13	±7.85
Paternal education/years	9.48	±4.3
Family income level		
Low	83	51.9
Middle	69	43.1
High	8	5
Parent filling in the scales		
Mother	178	67.4
Father	43	16.3
Other primary caregiver	43	16.3

SD, standard deviation; ID, intellectual disability.

**Table 2:** Clinical characteristics of participants.

	Mean / n	SD / %
ASD diagnosis through clinical evaluation based on DSM-5-TR		
Yes	45	23.1
No	150	76.9
Any other psychiatric disorder diagnosis through clinical evaluation based on DSM-5-TR		
Yes	70	30.6
No	159	69.4
Total T-score of DBC-P		
Little concern (T-score <40)	29	12.4
Moderate concern (T-score =40-50)	102	43.6
Serious concern (T-score >50)	103	44
DBC-P/Disruptive subscale T-score		
Little concern (T-score <40)	29	12.4
Moderate concern (T-score =40-50)	101	43.2
Serious concern (T-score >50)	104	44.4
DBC-P/Self-absorbed subscale T-score		
Little concern (T-score <40)	21	9
Moderate concern (T-score =40-50)	123	52.6
Serious concern (T-score >50)	90	38.5
DBC-P/Communication disturbance subscale T-score		
Little concern (T-score <40)	29	12.4
Moderate concern (T-score =40-50)	106	45.3
Serious concern (T-score >50)	99	42.3
DBC-P/Anxiety subscale T-score		
Little concern (T-score <40)	25	10.7
Moderate concern (T-score =40-50)	105	44.9
Serious concern (T-score >50)	104	44.4
DBC-P/Social relating subscale T-score		
Little concern (T-score <40)	49	20.6
Moderate concern (T-score =40-50)	74	31.1
Serious concern (T-score >50)	115	48.3
Total T-score of DBC-T		
Little concern (T-score <40)	20	9.9
Moderate concern (T-score =40-50)	98	48.5
Serious concern (T-score >50)	84	41.6
DBC-T/Disruptive subscale T-score		
Little concern (T-score <40)	26	12.9
Moderate concern (T-score =40-50)	94	46.5
Serious concern (T-score >50)	82	40.6
DBC-T/Self-absorbed subscale T-score		
Little concern (T-score <40)	0	0
Moderate concern (T-score =40-50)	127	62.9
Serious concern (T-score >50)	75	37.1
DBC-T/Communication disturbance subscale T-score		
Little concern (T-score <40)	36	17.8
Moderate concern (T-score =40-50)	82	40.6
Serious concern (T-score >50)	84	41.6
DBC-T/Anxiety subscale T-score		
Little concern (T-score <40)	28	13.9
Moderate concern (T-score =40-50)	86	42.6
Serious concern (T-score >50)	88	43.6
DBC-T/Social relating subscale T-score		
Little concern (T-score <40)	33	13.9
Moderate concern (T-score =40-50)	97	40.9
Serious concern (T-score >50)	107	45.1
Presence of comorbidity based on DBC-P		
Yes (total T-score ≥53)	68	29.1
No (total T-score <53)	166	70.9
Presence of comorbidity based on DBC-T		
Yes (total T-score ≥48)	93	46
No (total T-score <48)	109	54
ASD screening based on DBC-ASA		
Positive (Unweighted score ≥17)	69	37.9
Negative (Unweighted score <17)	113	62.1
Presence of comorbidity based on SDQ-P		
Yes (total score ≥17)	59	29.8
No (total score <17)	139	70.2
Presence of comorbidity based on SDQ-T		
Yes (total score ≥16)	69	31.7
No (total score <16)	149	68.3

SD, standard deviation; ASD, autism spectrum disorder; DBC-P, developmental behaviour checklist-parent form; DBC-T, developmental behaviour checklist-teacher form; SDQ-P, strengths and difficulties questionnaire-parent form; SDQ-T, strengths and difficulties questionnaire-teacher form.

The validation parameters in **Table 3** highlight the performance of the DBC-P, DBC-T, and DBC-ASA in detecting comorbidities, compared to the SDQ forms, and in detecting ASD, compared to SDQ forms clinical ASD diagnosis, respectively. The DBC-P achieved a correct classification rate of 80.6%. It demonstrated a sensitivity of 64.4%, indicating a moderate ability to identify individuals with comorbidities correctly. The specificity was higher at 87.6%, showing a strong ability to identify those without comorbidities correctly. The positive predictive value was 69.1%, indicating a 69.1% chance that participants identified with comorbidities had the comorbidities. The negative predictive value was 85.1%, suggesting that 85.1% of those identified as not having comorbidities were without them. The AUC was 0.76, signifying good overall accuracy. Additionally, the moderate Kappa coefficient of 0.53 reflects an acceptable level of agreement between observed and expected classifications. The detection rate was 19.4%, reflecting the proportion of actual cases correctly identified.

**Table 3:** Validation parameters of DBC parent and teacher forms and autism screening algorithm.

			SDQ-P Comorbidity				SDQ-T Comorbidity				Clinical ASD diagnosis	
		n	Yes	No		n	Yes	No		n	Yes	No
	DBC-P Comorbidity	Yes	38	17	DBC-T Comorbidity	Yes	52	31	DBC-ASA ASD screening	Yes	18	40
		No	21	120		No	6	97		No	13	73
Correct Classification Rate	% (95% CI)	0.806 (0.744-0.859)			% (95% CI)	0.801 (0.736-0.856)			% (95% CI)	0.632 (0.545-0.711)		
Sensitivity	% (95% CI)	0.644 (0.522-0.766)			% (95% CI)	0.897 (0.818-0.975)			% (95% CI)	0.581 (0.407-0.754)		
Specificity	% (95% CI)	0.876 (0.821-0.931)			% (95% CI)	0.758 (0.684-0.832)			% (95% CI)	0.646 (0.558-0.734)		
Positive Likelihood Ratio	% (95% CI)	5.190 (3.200-8.419)			% (95% CI)	3.702 (2.692-5.091)			% (95% CI)	1.640 (1.111-2.421)		
Negative Likelihood Ratio	% (95% CI)	0.406 (0.287-0.576)			% (95% CI)	0.137 (0.064-0.293)			% (95% CI)	0.649 (0.420-1.004)		
Positive Predictive Value (Precision)	% (95% CI)	0.691 (0.569-0.813)			% (95% CI)	0.627 (0.522-0.731)			% (95% CI)	0.310 (0.191-0.429)		
Negative Predictive Value	% (95% CI)	0.851 (0.792-0.910)			% (95% CI)	0.942 (0.897-0.987)			% (95% CI)	0.849 (0.773-0.925)		
Kappa	% (95% CI)	0.530 (0.399-0.661)			% (95% CI)	0.585 (0.471-0.700)			% (95% CI)	0.172 (0.021-0.324)		
Detection Rate	% (95% CI)	0.194 (0.139-0.249)			% (95% CI)	0.280 (0.215-0.345)			% (95% CI)	0.125 (0.071-0.179)		
AUC	% (95% CI)	0.760 (0.671-0.849)			% (95% CI)	0.827 (0.751-0.903)			% (95% CI)	0.613 (0.482-0.744)		

DBC-P, developmental behaviour checklist-parent form; DBC-T, developmental behaviour checklist-teacher form; DBC-ASA, developmental behaviour checklist-autism screening algorithm; SDQ-P, strengths and difficulties questionnaire-parent form; SDQ-T, strengths and difficulties questionnaire-teacher form; ASD, autism spectrum disorder; AUC, area under the curve; CI, confidence interval.

The DBC-T demonstrated slightly better performance, with a correct classification rate of 80.1%. The test showed a high sensitivity of 89.7%, and a specificity of 75.8%, though with a higher rate of false positives than the DBC-P. The AUC was 0.827, indicating strong predictive performance for detecting comorbidities based on the SDQ-T. Additionally, the Kappa coefficient was 0.585, indicating moderate agreement between observed and expected classifications, and the detection rate was 28%, suggesting it identified a meaningful proportion of actual cases. These findings underscore the validity and reliability of the DBC-P and DBC-T in identifying comorbid emotional and behavioral problems, with the teacher form performing particularly well.

The DBC-ASA for ASD screening yielded a lower correct classification rate of 63.2%, with a sensitivity of 58.1% and a specificity of 64.6%. The positive predictive value was 31%, meaning only 31% of those identified as having ASD were correctly diagnosed. The AUC was 0.613, suggesting moderate accuracy. The Kappa coefficient was 0.172, indicating slight agreement between observed and expected classifications, and the detection rate was 12.5%, highlighting a relatively low proportion of actual ASD cases correctly identified. The Cronbach’s alpha coefficient for the DBC-ASA was 0.911. The DBC-ASA shows moderate accuracy in screening for ASD.

**Table 4** indicates strong convergent validity between the DBC and SDQ. For the DBC-P, total scores correlated with the SDQ-P (r=0.704), disruptive scores with conduct problems (r=0.594), and hyperactivity/inattention (r=0.600), self-absorbed scores with hyperactivity/inattention (r=0.608), anxiety scores with emotional symptoms (r=0.533), and social relating scores with peer problems (r=0.411). For the DBC-T, total scores correlated with the SDQ-T (r=0.724), disruptive scores with conduct problems (r=0.559), and hyperactivity/inattention (r=0.649), self-absorbed scores with hyperactivity/inattention (r=0.672), anxiety scores with emotional symptoms (r=0.542), and social relating scores with peer problems (r=0.455). These results confirm that the consistency of the DBC forms with measures of comorbid emotional and behavioral issues is strong.

**Table 4:** Correlation parameters of DBC and SDQ for the parent and teacher forms.

r p	SDQ-P Total	Emotional symptoms	Conduct problems	Hyperactivity/ inattention	Peer problems	Prosocial behavior
DBC-P Total	*0.704* *<.001*	0.467 <.001	0.528 <.001	0.623 <.001	0.411 <.001	-0.110 .103
Disruptive	0.658 <.001	0.392 <.001	0.594 <.001	0.600 <.001	0.338 <.001	-0.068 .318
Self-absorbed	0.641 <.001	0.388 <.001	0.432 <.001	0.608 <.001	0.389 <.001	-0.141 .037
Communication disturbance	0.535 <.001	0.407 <.001	0.367 <.001	0.456 <.001	0.341 <.001	0.025 .717
Anxiety	0.561 <.001	0.533 <.001	0.360 <.001	0.454 <.001	0.302 <.001	0.020 .763
Social relating	0.527 <.001	0.457 <.001	0.387 <.001	0.375 <.001	0.412 <.001	-0.250 <.001
r p	SDQ-T Total	Emotional symptoms	Conduct problems	Hyperactivity/ inattention	Peer problems	Prosocial behavior
DBC-T Total	*0.724* *<.001*	0.425 <.001	0.473 <.001	0.620 <.001	0.451 <.001	-0.293 <.001
Disruptive	0.720 <.001	0.374 <.001	0.559 <.001	0.649 <.001	0.361 <.001	-0.236 .001
Self-absorbed	0.644 <.001	0.239 .001	0.469 <.001	0.672 <.001	0.358 <.001	-0.354 <.001
Communication disturbance	0.524 <.001	0.332 <.001	0.316 <.001	0.399 <.001	0.411 <.001	-0.210 .003
Anxiety	0.507 <.001	0.542 <.001	0.295 <.001	0.362 <.001	0.311 <.001	-0.109 .129
Social relating	0.539 <.001	0.426 <.001	0.251 <.001	0.388 <.001	0.455 <.001	-0.343 <.001

DBC-P, developmental behaviour checklist-parent form; DBC-T, developmental behaviour checklist-teacher form; SDQ-P, strengths and difficulties questionnaire-parent form; SDQ-T, strengths and difficulties questionnaire-teacher form.

The internal consistency reliability of the DBC forms is strong, as shown by a Cronbach’s alpha of 0.964 for the DBC-P and 0.961 for the DBC-T. Subscale alphas for the DBC-P include disruptive (0.914), self-absorbed (0.911), communication disturbance (0.769), anxiety (0.733), and social relating (0.700). For the DBC-T, the alphas were disruptive (0.908), self-absorbed (0.916), communication disturbance (0.751), anxiety (0.787), and social relating (0.788).

Interrater reliability displayed low to moderate correlations between parent and teacher ratings (See **Table 5**). Specifically, 43.1% of cases showed no comorbidities in common, while 16.1% had comorbidities identified by both. A significant difference between parent and teacher assessments highlights the need for multi-informant approaches (p<.001). Similar patterns were observed in the SDQ forms.

**Table 5:** Correlation parameters of the parent and teacher forms for DBC and SDQ.

r p	DBC-T Total	Disruptive	Self- absorbed	Communication disturbance	Anxiety	Social relating
DBC-P Total	*0.313* *<.001*	0.221 .009	0.319 <.001	0.216 .011	0.260 .002	0.323 <.001
Disruptive	0.255 .003	*0.241* *.005*	0.222 .009	0.173 .043	0.197 .021	0.239 .002
Self-absorbed	0.317 <.001	0.211 .013	*0.392* *<.001*	0.184 .032	0.202 .018	0.344 <.001
Communication disturbance	0.206 .016	0.131 .127	0.245 .004	*0.174* *.042*	0.157 .066	0.218 .004
Anxiety	0.148 .085	0.068 .428	0.118 .171	0.101 .242	*0.212* *.013*	0.170 .026
Social relating	0.237 .005	0.133 .117	0.225 .007	0.149 .080	0.190 .025	*0.322* *<.001*
r p	SDQ-T Total	Emotional symptoms	Conduct problems	Hyperactivity/ inattention	Peer problems	Prosocial behavior
SDQ-P Total	*0.369* *<.001*	0.242 .003	0.296 <.001	0.281 .001	0.183 .028	0.109 .188
Emotional symptoms	0.129 .119	*0.178* *.026*	0.162 .043	-0.020 .801	0.044 .586	-0.035 .667
Conduct problems	0.290 <.001	0.191 .016	*0.299* *<.001*	0.199 .012	0.151 .061	0.081 .310
Hyperactivity/ inattention	0.336 <.001	0.167 .037	0.244 .002	*0.382* *<.001*	0.191 .017	0.161 .043
Peer problems	0.244 .003	0.122 .131	0.097 .236	0.260 .001	*0.275* *.001*	0.208 .009
Prosocial behavior	0.234 .004	0.160 .045	0.152 .057	0.133 .095	0.218 .006	*0.241* *.002*

DBC-P, developmental behaviour checklist-parent form; DBC-T, developmental behaviour checklist-teacher form; SDQ-P, strengths and difficulties questionnaire-parent form; SDQ-T, strengths and difficulties questionnaire-teacher form.

Test-retest reliability over an average of one month showed high correlation coefficients of 0.856 for the DBC-P and 0.945 for the DBC-T, demonstrating excellent stability and robust test-retest reliability in the subscales (See **Table 6**). These results confirm that the DBC is a consistent and stable measure for monitoring emotional and behavioral changes over time.

**Table 6:** Correlation parameters of test and re-test applications for DBC parent and teacher forms.

r p	Re-test/ DBC-P Total	Disruptive	Self- absorbed	Communication disturbance	Anxiety	Social relating
Test/ DBC-P Total	*0.856* *<.001*	0.830 <.001	0.496 .085	0.527 .064	0.804 .001	0.573 .041
Disruptive	0.755 .003	*0.798* *.001*	0.392 .185	0.404 .170	0.735 .004	0.512 .074
Self-absorbed	0.654 .015	0.584 .036	*0.906* *<.001*	0.699 .008	0.410 .164	0.584 .036
Communication disturbance	0.874 <.001	0.847 <.001	0.776 .002	*0.821* *.001*	0.720 .005	0.424 .148
Anxiety	0.716 .006	0.692 .009	0.392 .185	0.456 .118	*0.958* *<.001*	0.592 .033
Social relating	0.382 .198	0.279 .355	0.343 .251	0.220 .470	0.471 .105	*0.844* *<.001*
r p	Re-test/ DBC-T Total	Disruptive	Self-absorbed	Communication disturbance	Anxiety	Social relating
Test/ DBC-T Total	*0.945* *<.001*	0.913 <.001	0.476 .139	0.797 .003	0.848 .001	0.477 .138
Disruptive	0.968 <.001	*0.989* *<.001*	0.554 .077	0.753 .007	0.747 .008	0.582 .060
Self-absorbed	0.715 .013	0.632 .037	*0.825* *.002*	0.512 .107	0.468 .146	0.587 .058
Communication disturbance	0.633 .037	0.552 .099	0.283 .400	*0.618* *.043*	0.833 .001	-0.045 .895
Anxiety	0.202 .552	0.168 .621	-0.227 .501	0.190 .576	*0.612* *.045*	0.028 .934
Social relating	0.095 .768	-0.082 .800	0.023 .943	0.154 .633	0.271 .393	*0.644* *.024*

DBC-P, developmental behaviour checklist-parent form; DBC-T, developmental behaviour checklist-teacher form.

The sociodemographic and clinical characteristics of children and adolescents with ID were compared, with a focus on distinguishing those with and without psychiatric comorbidities through clinical evaluation based on DSM-5-TR (See **Table 7**). Findings show significant differences in the duration of special education (p=.040) and maternal education (p=.013), with more extended education in the psychiatric comorbidity group. Clinical assessments using the DBC and SDQ forms revealed higher scores in almost all domains in children and adolescents with psychiatric comorbidity, indicating more significant behavioral and emotional difficulties. When all these are considered together, the DBC forms differentiate between the groups with and without psychiatric comorbidities.

**Table 7:** Comparison of the sociodemographic and clinical characteristics of children and adolescents with intellectual disability diagnosed with any psychiatric comorbidity through clinical evaluation based on DSM-5-TR with those without psychiatric comorbidity.

	Children and adolescents with psychiatric comorbidities		Children and adolescents without psychiatric comorbidities		
	Mean / n	SD / %	Mean / n	SD / %	p value
Age/years	13	±4	12	±4	.163
Sex					.618
Female	24	34.3	60	37.7	
Male	46	65.7	99	62.3	
Special education/years	7.41	±4.67	5.95	±3.85	*.040*
ID level					.077
Mild	39	60.9	110	67.8	
Moderate	13	20.3	24	16.4	
Severe	12	18.8	12	8.2	
Maternal age/years	40.61	±7.66	40.6	±7.65	.906
Maternal education/years	9.65	±4.17	7.83	±4.28	*.013*
Paternal age/years	44.11	±7.69	43.69	±7.72	.597
Paternal education/years	10.24	±4.09	9.02	±4.39	.107
Family income level	7463.33	±5682.14	6081.41	±3579.59	.493
ASD diagnosis					.444
Yes	14	25.9	27	20.8	
No	40	74.1	103	79.2	
DBC-ASA	18.94	±10.66	12.31	±11.23	*<.001*
DBC-P/Total	54.58	±9.27	48.08	±9.9	*<.001*
DBC-P/Disruptive	55.12	±9.96	47.93	±9.52	*<.001*
DBC-P/Self-absorbed	54.06	±10.48	48.36	±9.53	*<.001*
DBC-P/Communication disturbance	53.84	±9.23	48.39	±9.77	*<.001*
DBC-P/Anxiety	52.87	±9.51	48.71	10.33	*.001*
DBC-P/Social relating	53.06	±9.83	48.52	±9.9	*.001*
DBC-T/Total	56.17	±12.48	48.54	±8.59	*.001*
DBC-T/Disruptive	56.06	±12.88	49.05	±8.65	*.004*
DBC-T/Self-absorbed	56.46	±14.03	48.25	±7.63	*<.001*
DBC-T/Communication disturbance	53.96	±10.41	48.97	±9.41	*.004*
DBC-T/Anxiety	54	±11.67	49.4	±9.56	*.029*
DBC-T/Social relating	54.15	±9.83	48.49	±10.07	*<.001*
SDQ-P/Total	17	±6	13	±6	*<.001*
SDQ-P/Emotional symptoms	3	±2	2	±2	*.008*
SDQ-P/Conduct problems	3	±2	2	±2	*.001*
SDQ-P/Hyperactivity and inattention	7	±3	5	±2	*<.001*
SDQ-P/Peer problems	5	±2	4	±2	*<.001*
SDQ-P/Prosocial behavior	6	±3	5	±3	.394
SDQ-T/Total	17	±7	12	±5	*<.001*
SDQ-T/Emotional symptoms	4	±3	2	±2	*<.001*
SDQ-T/Conduct problems	3	±2	1	±1	*.005*
SDQ-T/Hyperactivity and inattention	7	±3	5	±2	*<.001*
SDQ-T/Peer problems	4	±2	4	±2	.051
SDQ-T/Prosocial behavior	4	±3	5	±3	.122

DBC-P, developmental behaviour checklist-parent form; DBC-T, developmental behaviour checklist-teacher form; DBC-ASA, developmental behaviour checklist-autism screening algorithm; SDQ-P, strengths and difficulties questionnaire-parent form; SDQ-T, strengths and difficulties questionnaire-teacher form; ASD, autism spectrum disorder.

## 5 Discussion

This study aimed to evaluate the validity and reliability of the Turkish version of the revised DBC forms in identifying emotional and behavioral problems in children and adolescents with ID in Turkey. The findings provide robust evidence for the psychometric soundness of the DBC-P and DBC-T in detecting this population’s emotional, behavioral, and social difficulties. Both forms demonstrated strong reliability and validity, particularly when compared with the SDQ, a commonly used tool for assessing emotional and behavioral functioning across various mental health domains. However, the DBC-ASA showed moderate accuracy in screening for ASD, with sensitivity and specificity levels below the desired thresholds. While the DBC-ASA provides valuable insights into autism-related behaviors, its performance suggests it is best utilized as an initial screening tool, alongside other diagnostic instruments. These results underscore the value of the DBC in assessing psychiatric comorbidities in Turkish children and adolescents with ID, although further refinement is needed for more effective ASD screening.

Studies on the prevalence of mental disorders in children and adolescents with ID have shown co-occurrence rates ranging from 30% to 50%, depending on the study population. The risk of mental disorders is approximately 2.8 to 4.5 times higher in this group than in the general population (25). Consistent with these findings, a meta-analysis of 19 studies involving 6,151 children and adolescents revealed that the prevalence of psychiatric symptoms, as measured by the DBC, was 38%, regardless of ID severity (26). Diagnoses such as ADHD (39%), anxiety disorders (7–34%), conduct and externalizing disorders (3–21%), and depressive disorders (3–5%) were similar to those in community samples, except for ADHD (26). However, prevalence estimates based on categorical diagnoses using systems such as the International Classification of Diseases, 11th Revision (ICD-11) or DSM-5-TR may not fully capture the burden of emotional and behavioral problems in this population due to their unique manifestations and the communication challenges associated with the ID (27). Emphasizing specific symptom clusters reflected in the DBC subscales may better represent the mental health challenges faced by this group (5). The DBC forms may also identify subclinical cases that require intervention but do not meet formal diagnostic criteria under DSM-5-TR.

Studies on ASD prevalence in children with ID have reported rates between 4% and 28%, often attributed to methodological differences, with one study reporting a prevalence of 18.04% (28). Our sample consisted of randomly selected children with parental consent; the clinically evaluated ASD diagnosis rate was 23.1%. This rate and findings on other psychiatric comorbidities suggest that our sample reflects naturalistic trends reported in the literature. Given the high comorbidity of ASD with ID, effective screening for ASD in this population is critical. Missing an ASD diagnosis can result in delayed interventions and poorer psychosocial and family functioning. We identified 37.9% of children with ID as being at risk for ASD using the DBC-ASA in our sample. However, the sensitivity (58%) and specificity (65%) obtained in this study at the cut-off value of 17 were lower than those reported in the original study (86% and 69%, respectively) (23). A cross-validation study between German and Swedish cultures found similar specificity (63%) but higher sensitivity (79%) at the same cut-off (24). A UK study reported sensitivity and specificity values of 95% and 42%, respectively, at the same threshold (29). These variations across populations highlight the influence of cultural and contextual factors on the DBC-ASA’s performance. Differences in how parents and teachers perceive and report ASD-related behaviors, as well as variations in clinical diagnostic criteria and healthcare access, may contribute to these discrepancies. A deeper exploration of these cultural influences could provide valuable insights into adapting ASD screening tools for diverse populations. In our sample, children with mild ASD features may have been overlooked because of fewer or masked behavioral problems, resulting in false negatives (30, 31). Since some children with ASD, particularly those with mild or masked symptoms, may not meet the DBC-ASA threshold, clinicians should consider additional screening tools and structured clinical observations. Although it has lower sensitivity, the Turkish version of the DBC-ASA is still considered an acceptable screening tool because it is affordable and useful in large population studies. This can facilitate further evaluations and reduce the financial burden of ASD diagnosis and intervention in children and adolescents with ID. Its affordability makes it particularly useful in low-resource environments, where access to comprehensive diagnostic assessments may be limited. However, given its moderate sensitivity, it should not be used as a standalone diagnostic tool. Instead, integrating it with other screening measures or clinical evaluations could enhance its effectiveness in identifying children with ASD who might otherwise go unnoticed. Incorporating teacher and parent interviews, as well as standardized diagnostic assessments, could improve accuracy and ensure that children receive appropriate interventions.

The validation analyses of the DBC-P and DBC-T were based on clinical cut-off scores from the US sample rather than the original Australian sample (13). This decision was informed by the recency of the US sample and its similarities to our study population in terms of treatment and care access (14). The SDQ was used as a reference scale due to the established good agreement between the SDQ and DBC forms for children with ID (32). In the original study, the DBC-P had a sensitivity of 83%, specificity of 85%, and an AUC of 92% at a cut-off of 44 (13). The Turkish version of the DBC-P, with a cut-off of 53, had a comparable specificity (88%) but lower sensitivity (64%) and AUC (76%). These discrepancies may reflect cultural differences in reporting behavioral problems or the influence of factors such as parental education and duration of special education. At a cut-off of 48, the DBC-T demonstrated a sensitivity of 90% and specificity of 76%, thereby outperforming the DBC-P. Although its AUC (83%) was lower than the original study’s 93% when using a cut-off of 30, it remained robust (33). Differences in parent and teacher reports, including teachers’ higher frequency of reported problems, likely reflect contextual variations in behavior across home and school environments (15, 19, 34).

The study has limitations, including reliance on self-reported data, which may be influenced by subjective perceptions and cultural factors, particularly given the low educational levels of some parents. The differences between teacher and parent reports emphasize the importance of using multiple informants for the DBC-P and DBC-T forms. Additionally, the absence of a gold-standard tool like the Autism Diagnostic Observation Schedule (ADOS) limits the validation of ASD traits alongside the DBC-ASA results. The variability in the DBC-ASA performance across populations and the observed sensitivity and specificity differences underline the influence of cultural and contextual factors. Lastly, while using different cut-off points based on intellectual levels is suggested in the literature, our analyses did not reveal significant differences, supporting a single cut-off for simplicity.

## 6 Conclusion

The study is valuable and can impact several domains, particularly for clinicians, educators, researchers, and policymakers in Turkey and other poor-resource countries with similar cultural contexts, by addressing critical gaps in assessing and understanding emotional and behavioral problems in children and adolescents with ID.

These domains include 1) Improved Diagnostic and Clinical Practice; the validation of the DBC-P and DBC-T provides clinicians with accessible, reliable, and culturally adapted tools to identify emotional and behavioral difficulties in children and adolescents with ID. The findings underscore the DBC’s ability to recognize various emotional and behavioral challenges, including subclinical cases often overlooked by categorical diagnostic frameworks like DSM-5-TR and ICD-11. This ensures that children requiring support are not neglected because their difficulties fall outside strict diagnostic criteria. 2) Tailored Interventions: Early detection of emotional, behavioral, and social difficulties is essential for implementing timely interventions. These interventions can greatly enhance developmental outcomes and lessen the long-term impact of untreated psychiatric comorbidities. The DBC’s subscale-based insights provide a clearer understanding of specific symptom clusters, enabling clinicians and educators to create tailored interventions that address each child’s unique needs. 3) Screening for ASD: While the DBC-ASA demonstrated moderate sensitivity and specificity in this study, it remains a cost-effective and valuable tool for initial ASD screening in children with ID. This is particularly important in settings with limited resources, where comprehensive diagnostic evaluations may not always be possible. By identifying children at risk of ASD early, the DBC-ASA can help mitigate the risks associated with delayed diagnoses, such as missed opportunities for early intervention, which are critical for improving outcomes in children with ASD. 4) Cultural and Contextual Relevance: The study highlights the importance of cultural adaptation in psychological assessment tools. The variations in sensitivity, specificity, and reporting patterns (e.g., between parents and teachers) demonstrate the necessity of considering cultural norms, parental education levels, and contextual factors for understanding behavior. The validated Turkish DBC can serve as a model for adapting and validating similar tools in other non-Western settings, where the lack of culturally appropriate assessments often impedes careful evaluation. 5) Advancing Research: The availability of a validated Turkish DBC supports large-scale epidemiological studies aimed at better understanding the prevalence and characteristics of psychiatric comorbidities in children with ID. Such studies can inform public health strategies and guide resource allocation. This study establishes the foundation for cross-cultural comparisons of emotional and behavioral problems in children with ID, facilitating global collaborations and offering a deeper understanding of the interplay between culture, behavior, and mental health. 6) Policy Implications: As demonstrated in Australia, where the DBC was initially developed (12), the findings highlight the urgent need for increased resources to support children and adolescents with ID in Turkey. Policymakers can use this evidence to prioritize funding for mental health services, early intervention programs, and special education initiatives. The study underscores the importance of developing tools and interventions that address the diverse needs of children with ID, promoting their inclusion in educational and social settings. 7) Training and Capacity Building: The validated DBC can be integrated into training programs for clinicians, educators, and mental health professionals in Turkey. This ensures they are equipped with practical tools and knowledge to assess and meet the needs of children with ID. By providing a strong evidence base, the study contributes to raising awareness about the mental health challenges faced by children and adolescents with ID, encouraging a more empathetic and informed approach among professionals and caregivers. 8) Cost-Effectiveness: The DBC’s low cost makes it an ideal tool for widespread use, especially in resource-constrained settings. Its utility in identifying at-risk cases can alleviate the financial burden associated with delayed diagnoses and untreated mental health conditions, benefiting families, schools, and healthcare systems.

Finally, the study’s findings hold implications beyond the Turkish context. By highlighting the significance of culturally adapting and validating assessment tools, this approach establishes a benchmark for enhancing mental health services for children with intellectual disabilities in various settings. Furthermore, the study stresses the necessity for continued research to refine these tools, ensuring they remain relevant and helpful across different populations. The Turkish DBC has the potential to significantly improve the quality of care and support for children and adolescents with ID, promoting better developmental, educational, and psychosocial outcomes.

## Data Availability

The raw data supporting the conclusions of this article will be made available by the authors, without undue reservation.
